# Antibacterial Action, Substantivity and Anti-plaque Effect of Different Toothpaste Slurries – A Randomised Controlled Trial

**DOI:** 10.3290/j.ohpd.b2182977

**Published:** 2021-10-22

**Authors:** Nicole Birgit Arweiler, Friederike Müller-Breitenkamp, Christian Heumann, Oliver Laugisch, Thorsten M. Auschill

**Affiliations:** a Professor, Director and Head, Department of Periodontology and Peri-Implant Diseases, Dental School and Hospital, Philipps-University of Marburg, Germany. Principal investigator, conception, design and management of the study, wrote the manuscript.; b Dentist, Department of Periodontology and Peri-Implant Diseases, Dental School and Hospital, Philipps-University of Marburg, Germany. Performed the experiments in partial fulfilment of requirements for a Dr. med. dent. degree.; c Statistician, Professor, Department of Statistics, Ludwig-Maximilians-University, Munich, Germany. Contributed to and performed statistical evaluation of all data.; d Research Honorary, Department of Periodontology and Peri-Implant Diseases, Dental School and Hospital, Philipps-University of Marburg, Germany. Contributed essentially to the discussion, wrote the manuscript.; e Professor, Department of Periodontology and Peri-Implant Diseases, Dental School and Hospital, Philipps-University of Marburg, Germany. Contributed substantially to conception and design of the study as well as to the discussion, proofread the manuscript.

**Keywords:** anti-plaque agents, biofilm vitality, chemical plaque control, substantivity, toothpaste

## Abstract

**Purpose:**

This single-center, clinically controlled, double-blinded, randomised, crossover study aimed to evaluate and compare the antibacterial effect, substantivity and patients’ acceptance of three toothpaste slurries after a single application on established biofilms observed for 24 h.

**Materials and Methods:**

Twenty-four participants started a test cycle after refraining from oral hygiene for 48 h, with a baseline plaque sample measuring biofilm vitality (in %; VF_0_) using vital fluorescence (VF). They were instructed to rinse for 1 min with either an amine fluoride, stannous chloride (ASC), an herbal (SBC) or a sodium fluoride (SFL) toothpaste prepared as slurries. Every two hours up to 12 and after 24 h, plaque samples were harvested (VF_2_-VF_24_%). Plaque-covered areas (PA in %) were evaluated after 24 h using digital photographs. Patients’ acceptance was determined by visual analogue scale (VAS) questionnaire.

**Results:**

All participants (16 women, 8 men; 27.5 ± 7.9 years) completed all cycles. Two hours after application (VF2), all toothpastes showed a statistically significant reduction in bacterial vitality (p < 0.05), maintained up to 12 h. ASC revealed statistically significantly lower vitality values compared to SBC at VF_2_, VF_4_, VF_8_, VF_12_ and VF_24_, and at VF_4_, VF_12_ and VF_24_ compared to SFL (p < 0.05), while SBC and SFL did not differ statistically significantly at any time point. The preferred toothpastes were SFL (18/24 participants) and ASC (15/24 participants).

**Conclusions:**

All toothpastes showed statistically significant anti-plaque effects on established plaque biofilm and a substantivity up to 24 h compared to their baseline, while ASC still presented a statistically significant effect after 12 and 24 h compared to SBC and SFL.

Pathogens organised in dental biofilms are the main cause of inflammation and destruction of tooth-supporting structures.^23^ In order to prevent disease initiation and progression, its regular daily mechanical removal with toothbrush and toothpaste is indispensable.^10^ Antibacterial agents in toothpastes, such as metal ions, amine fluoride/stannous fluoride, essential oils, stannous fluoride with sodium hexametaphosphate (ZnF/SHMP) or triclosan/copolymer have been shown to be effective in inhibiting dental biofilms.^7,13,25^

While an immediate anti-plaque effect of antibacterial agents incorporated in toothpaste is desirable, it is not essential, since toothbrushing already removes the biofilm. However, since plaque bacteria accumulate again on teeth and the gum line after toothbrushing and form a dental biofilm, it is important that antibacterial agents adsorb and maintain their antimicrobial activity beyond the time of application. Thus, they should prevent bacterial recolonisation, kill bacteria or inhibit their growth until the next tooth cleaning, as well as affect remaining established plaque biofilm that could not be removed by toothbrush (or interdental cleaning). This retention or ‘substantivity’ of the antimicrobial substance, defined as slow release in an active status, is extremely important and should be tested in the whole toothpaste formulation, since inactivation in a complex toothpaste could nullify substantivity.

Substantivity can be assessed by the quantity of active substances that remain in the oral cavity,^15-17^ the effect on salivary bacteria,^1,2,12^ or by using a vital fluorescence technique that determines the vitality status of the dental biofilm by measuring the percentage of vital bacteria.^6,9^

In 2018, a new toothpaste was introduced containing a combination of amine fluorides (Olaflur) and stannous chlorides as active substances. This formulation yielded more pronounced plaque reduction compared with a monofluorophosphate toothpaste.^19^

While tin salts (e.g. stannous chlorides and stannous fluorides) have been known to be effective against oral bacteria since 1884,^20^ stannous chlorides were only seldom used in toothpaste formulations for their plaque inhibiting properties,^11,14,26^ but rather for their anti-erosion effects.^18,31^ The combination of stannous fluorides with silica or sodium hexametaphosphate are more often used, since they form insoluble stannous-rich complexes that precipitate in the outermost dentin or enamel layers^29^ and thus have desensitising effects.^27,28^

After a previous study^8^ compared the substantivity of toothpastes containing a combination of amine fluoride/stannous fluoride with a toothpaste containing herbal ingredients/sodium bicarbonate (as a benchmark toothpaste) and sodium fluoride only (as a standard control toothpaste) as well as with a chlorhexidine mouthrinse as the gold standard for highest substantivity, it became necessary to test the newly formulated combination of amine fluorides/stannous chlorides (instead of stannous fluorides) based on the same design and comparing with the same benchmark and control toothpastes as before.

Thus, the aim of the study was to evaluate the direct antibacterial effect, the substantivity of this effect, and patient’s acceptance of a newly formulated toothpaste combination of amine fluoride/stannous chloride as compared to two previously tested toothpastes applied as slurries and over an observation period of 24 h.

The working hypothesis was that the newly developed toothpaste formulation shows antibacterial effects, high substantivity and patients’ acceptance comparable to commercial toothpastes.

## Material and Methods

### Study Site and Experimental Design

**Fig 1:**
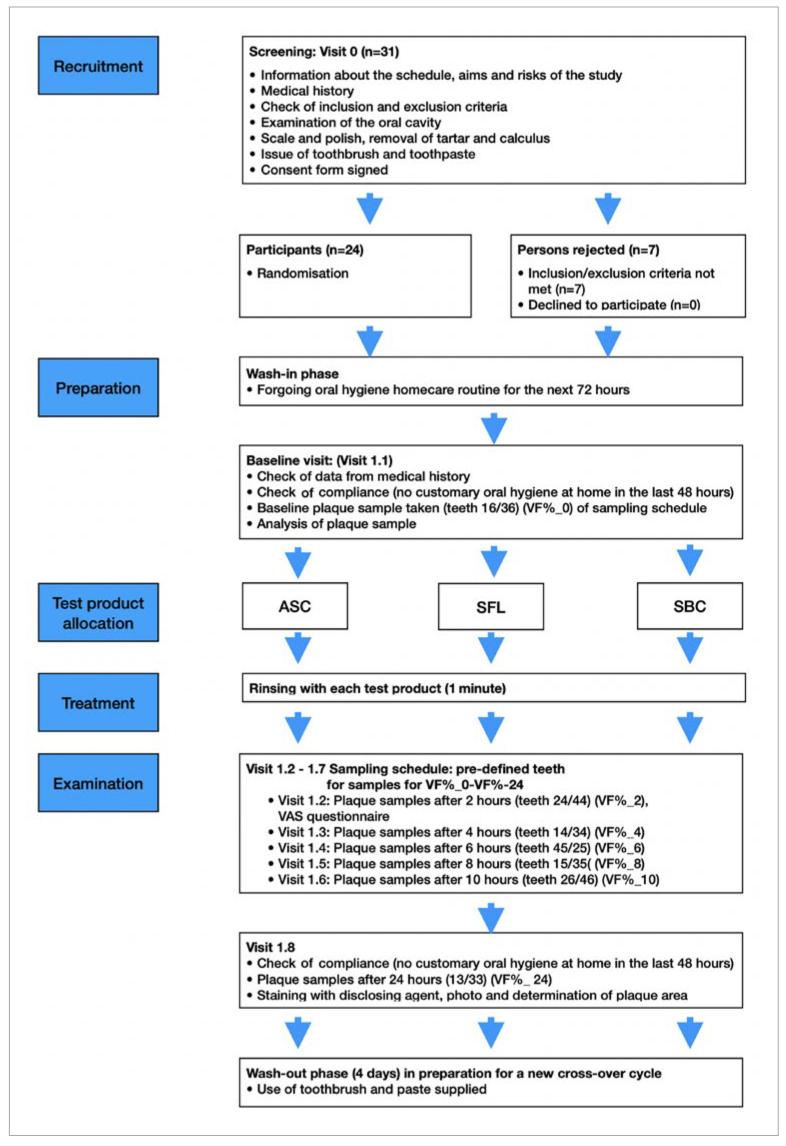
Study flowchart including the sampling schedule: pre-defined teeth for plaque samples timepoints (VF_0_%-VF_24_%).

The study was registered in the Trial Register of Clinical Studies (www.drks.de; DRKS00023738) and carried out at the Department of Periodontology and Peri-Implant Diseases, Philipps University of Marburg (UKGM), Germany. All lab-based examinations were performed in the department’s laboratory. The study protocol was approved by the Medical Ethics Committee, Philipps University of Marburg (#120/16) and conducted in accordance with the latest edition of the ethical principles of the Declaration of Helsinki, local laws, and the principles of Good Clinical Practice (GCP). STROBE guidelines were strictly followed. Upon written informed consent, 31 potential test subjects were screened, of whom 24 were included in this single-center, clinically controlled, double-blinded, randomised, crossover study as long as they met all in- or exclusion criteria. The study flowchart is presented in Fig 1.

### Study Population

Participants had to be between the ages of 18 and 70 years and in good health. They had to have at least 20 natural teeth (excluding wisdom teeth), no crowns or large restorations on incisors, confirm effective contraception, be non-smokers or former smokers for at least one year or occasional smokers with a maximum of 5 cigarettes per week. All participants needed to be willing not to use interdental cleaning devices that contained antibacterial agents, such as amine fluoride, chlorhexidine, silver ions etc, for the duration of the study.

The exclusion criteria were as follows: 1. severe systemic diseases (e.g. tumours, infectious diseases) and diseases that require regular systemic drugs (e.g. anti-hypertensives/Ca-antagonists); 2. the use of systemic antibiotics during the last 3 months or local antimicrobials that may affect the plaque biofilm (antibacterial mouth rinses, e.g. chlorhexidine, 4 weeks or less prior recruitment); 3. severe oropharyngeal infections, relevant dental disorders, ongoing dental treatment or any other medical treatment of the oral cavity; 4. any known allergy to previously used oral hygiene products and/or oral therapeutic agents and/or dental materials or a known allergy to any of the ingredients of the study products or standard toothpastes (e.g. Limonene), used during the study and the wash-out periods; 5. participants with signs of active periodontitis according to Armitage,^5^ or non-physiological tooth mobility, any other pathological change of the oral mucosa or gingiva (including gingivitis), poor oral hygiene (papilla bleeding index PBI > 30%) or carious lesions; 6. chronic drug abuse (alcohol, etc) or any other illness which does not allow the person to assess the nature and/or possible consequences of the study; 7. participation in another clinical study within the previous 30 days or parallel to this study; 8. women who were pregnant or breastfeeding.

### Test Products

- Test product (ASC): toothpaste with amine fluoride/stannous chloride, Caprylyl-Glycol, Phenylpropanol, 1400 ppm fluoride (meridol Parodont Expert toothpaste, Colgate Palmolive Europe; Therwil, Switzerland)
- Comparative product (SBC): toothpaste with herbal ingredients (Ratanhia root extract, purple coneflower extract, chamomile leaf, flower extract, myrrh extract, sage extract), sodium bicarbonate and sodium fluoride, 1400 ppm fluoride (Parodontax Fluorid, GlaxoSmithKline; Munich, Germany)
- Control product and wash-in/wash-out product (SFL): toothpaste with sodium fluoride, 1450 ppm fluoride (Signal Caries Protection, Unilever; Hamburg, Germany)

All toothpastes were applied as slurries in order to assess only the antibacterial effect and prevent confounding factors associated with mechanical tooth cleaning.

### Randomisation and Blinded Supply of the Products

All test products were delivered in identical packages (marked with numbers) and provided by a laboratory assistant who was not involved in the study. The study was performed under triple blind conditions. Neither the statistician (CH) nor the participants or examiner (FMB) knew which product was tested. Due to the cross-over design, every subject used all three study toothpastes in three cycles determined by a randomisation plan provided by the GMP-Manager of Colgate Palmolive and not accessible by study staff. Knowledge of the randomisation list was limited to the person involved in slurry preparation until unblinding the study. Participants were pseudonymised using numbers according to the order of participation in the initial test.

### Study Protocol

A flow chart of the study is shown in Fig 1. At the beginning of the study, participants received professional toothcleaning in order to obtain comparable plaque-free baseline conditions. A toothbrush (Meridol, Colgate Palmolive Europe; Therwil, Switzerland) and toothpaste preferably with a low concentration of active substances (Signal Caries Protection, Unilever; Hamburg, Germany) were handed out to each participant for mandatory use in a oral hygiene routine during a wash-in phase at the start of the study as well as the wash-out phases (4 days) between the three cycles.

After a wash-in period of 72 h, participants were instructed to refrain from their oral hygiene routine for 2 days (48 h) to allow dental biofilm growth.

At the beginning of each test cycle, a dental biofilm sample was obtained according to a sampling schedule (Fig 1) from two pre-defined teeth. A sterile dental probe (EXS 9, Hu-Friedy; Chicago, IL, USA) was used to obtain the biofilm samples (48-h biofilm) taken from the buccal aspects of the assigned teeth. Vitality value of biofilm bacteria was determined using vital fluorescence microscopy and served as baseline (VF_0_ in %).^8,25^ Then, participants were instructed to rinse with their allocated and prepared toothpaste slurry for 1 min under supervision of a lab assistant not otherwise involved in the study. Homogeneous slurries were mixed using 3 g toothpaste and 10 ml distilled water. Every two hours, further biofilm samples were harvested from two pre-defined teeth each up to 12 h after rinsing and again analysed using the vital fluorescence technique (VF_2_-VF_12_ in %). A final plaque sample was taken 24 h after rinsing (VF_24_ in %). At this time point, maxillary and mandibular incisors that had been omitted from VF analysis (Fig 1) were stained with plaque disclosing solution (Mira-2-Ton, Hager & Werken; Duisburg, Germany), and pictures were taken to assess plaque area (PA in %) in order to visually evaluate how toothpaste ingredients interact on established plaque.

Directly after rinsing, participants were asked to fill out a questionnaire to keep records on possible side effects and overall acceptance (e.g. taste). Adverse events were documented at each visit.

Before starting a new cycle, test subjects were asked to perform personal oral hygiene using the toothbrush and toothpaste provided (wash-out phase) for the next four days. During the following test cycles, the other two products followed the same protocol.

### Microbiological Evaluation: Biofilm Vitality (VF%) as the Primary Outcome Measure

Determination of VF% followed validated fluorescence microscopy used on each biofilm sample.^8,24,25^ After mounting the samples on a microscope slide (Thermo Scientific, Menzel; Braunschweig, Germany), they were coated with 5 μl of prepared staining solution and mixed carefully. After 2 min reaction time, samples were covered with a coverslip to exclude air. The VF technique is able to stain and differentiate between green (metabolically active) and red (metabolically inactive) bacteria. Four digital images of the stained biofilm were made under the fluorescent microscope (Axio Imager A2) with a camera (AxioCamHRc) and evaluated using the Axio Vision Rel. 4.8 software program (all Zeiss; Göttingen, Germany). The software calculates the proportion of green pixels compared to the sum of red and green pixels as the biofilm vitality in percent (VF in %) of each plaque sample. A total of 2304 digital vital fluorescence images were assessed in the three test cycles (24 test subjects x 3 test products x 8 measurements x 4 images).

### Plaque Area (PA%)

Plaque area was evaluated 24 h after rinsing and staining the biofilm covering the buccal surfaces of maxillary and mandibular incisors with a disclosing agent (Mira-2-Ton, Hager & Werken). Photographs were taken. PA% was then calculated as the percentage of covered area compared to the entire tooth area using a computer program (Axio Vision Rel. 4.8, Zeiss).

### Quality of Life Questionnaire and Patient Acceptance

Two hours after rinsing with each toothpaste slurry, questionnaires that also used visual analogue scales (VAS; 1-10) were filled out to subjectively assess patients’ acceptance, perceived taste, and ‘feeling of freshness’ of each test product and irritation.

### Sample Size, Power Calculation and Statistical Analysis

Before the start of the study, sample size was estimated, referring to a similar, earlier study.^3^ N=21 volunteers was calculated for a two-sided test of equality of means at the 0.05 level of significance with 80% power. Considering possible drop-outs, the sample size for the study was increased to 24 participants.

Pseudonymised data were collected in case report forms and later transferred to an Excel (Microsoft; Redmond, WA, USA) table using anonymisation. A statistician (CH) carried out the whole statistical analysis using SPSS v 23 (IBM; Armonk, NY, USA). Besides a descriptive analysis (mean, standard deviation, box plots, line diagrams), results of each treatment group at each timepoint (2, 4, 6, 8, 10, 12, 24 h) were compared with baseline (0 h) using the t-test for two dependent samples (intra-group comparisons), as these were observations on the same test subject. A linear mixed model for crossover design with subjects as random effects and the three treatment groups as fixed effects was used to calculate the differences in mean reductions (reduction from baseline [0 h] to 2, 4, 6, 8, 10, 12, 24 h) of the vital fluorescence (VF%) between the three treatment groups followed by pairwise comparisons. For each time point, a separate model was estimated. Biofilm vitality (VF%) at different timepoints were primary endpoints, whereas PA% and the questionnaire (only descriptive analysis) were secondary endpoints.

### Study Monitoring

Colgate Palmolive Europe monitored the study in accordance with international ethics and scientific quality standards (Good Clinical Practice directive).

## Results

In total, 24 participants (16 women, 8 men) between 20 and 55 years of age (mean: 27.5 ± 7.9 years) were included, signed a declaration of consent and completed all three test cycles.

### Biofilm Vitality (in %) and Substantivity

Two hours after application, all examined products showed a statistically significant reduction in VF_2_ compared to their baseline (VF_0_). ASC demonstrated the highest reduction (30%; p = 0.008), whereas SBC (16%) and SFL (23%) still showed statistically significant (< 0.001) but not such large differences (Table 1).

**Table 1:** Vitality values (VF in %, ± standard deviation) at baseline (VF_0_) and at different time points (VF_2_-VF_24_) as well as statistical comparisons to baseline

	VF_0_	VF_2_	P (VF_2_-VF_0_)	VF4	P (VF_4_-VF_0_)	VF6	P (VF_6_-VF_0_)	VF8	P (VF_8_-VF_0_)	VF10	P (VF_10_-VF_0_)	VF12	P (VF_12_-VF_0_)	VF24	P (VF_24_-VF_0_)
ASC	81.97	51.86	< 0.001	52.93	< 0.001	63.39	< 0.001	61.48	< 0.001	69.91	< 0.001	53.17	< 0.001	59.82	< 0.001
	± 8.95	±13.10	***	± 14.26	***	± 16.14	***	± 14.69	***	± 13.29	***	± 18.18	***	± 15.35	***
SFL	86.25	63.18	< 0.001	70.35	< 0.001	70.04	< 0.001	75.95	0.004	69.05	< 0.001	69.73	< 0.001	76.07	0.002
	± 8.94	± 14.78	***	± 14.21	***	± 10.82	***	± 10.66	**	± 11.60	***	± 13.68	***	± 11.98	**
SBC	81.48	64.91	< 0.001	71.01	0.008	74.69	0.006	73.61	0.026	70.98	0.008	67.28	< 0.001	77.36	**0.146**
	± 10.02	± 13.24	***	± 14.89	**	± 11.00	**	± 14.49	*	± 15.57	**	± 14.47	***	± 10.50	**n.s.**

n.s.: not significant (**bold**); *p ≤ 0.05; **p ≤ 0.01; ***p ≤ 0.001 (t-test for dependent samples).

Compared to baseline, a prolonged effect (substantivity) could be seen up to 24 h (VF_24_) except for SBC, which did not reach statistical significance (p > 0.05) at this final time point.

When comparing groups, ASC demonstrated statistically significantly lower vitality values compared to SBC and SFL at VF_2_, VF_4_, VF_8_, VF_12_ and VF_24_ (p < 0.05). After 10 h, the VF% of none of the test products varied significantly. VF_12_ and VF_24_ of ASC showed significantly lower values when compared to SBC and SFL. No statistically significant differences between SBC and SFL were detectable (Table 2).

**Table 2:** Statistical comparison of differences (changes from 0 h to all other time points 2, 4, 6, 8, 10, 12, 24 h) between the groups at the different time points

	pVF2	pVF4	pVF6	pVF8	pVF10	pVF12	pVF24
ASC vs SBC	0.004 **	0.001 **	0.022 *	0.041 *	1.000 n.s.	0.014 *	<0.001 ***
ASC vs SFL	0.251 n.s.	0.031 *	1.000 n.s.	0.132 n.s.	0.677 n.s.	0.049 *	0.018 *
SBC vs SFL	**0.329** **n.s.**	**0.827** **n.s.**	**0.089** **n.s.**	**1.000** **n.s.**	**0.350** **n.s.**	**1.000** **n.s.**	**0.452** **n.s.**

n.s.: not significant (**bold**); *p ≤ 0.05; **p ≤ 0.01; ***p ≤ 0.001 (linear mixed model for crossover design).

### Plaque Area (in %)

Plaque area revealed values of 18.89 ± ±13.68 % (ASC), 19.32 ± 14.23 % (SBC) and 20.76 ± 16.9 % (SFL), which were not significantly different from each other (p > 0.05).

### Quality of Life (Questionnaire)

Taste of SBC, SFL and ASC exhibited VAS values of 2.00 ± 1.79, 7.71 ± 1.81 and 6.71 ± 2.60 (0=unpleasant to 10 = pleasant) and a feeling of freshness was rated with 4.79 ± 2.99, 7.21 ± 1.98 and 6.50 ± 2.34, respectively (0 = not fresh at all to 10 = very fresh). An irritation of taste was reported in the SBC and ASC groups by 5 participants, but by none in the SFL group. While 18 subjects would agree to use SFL and 15 to use ASC on a regular basis, only 3 would consider SBC (Table 3).

**Table 3:** Evaluation of the questionnaire

	ASC	SFL	SBC
VAS value The taste of the product was… 0 = unpleasant…10 = pleasant	6.71 ± 2.6	7.71 ± 1.81	2.00 ± 1.79
VAS value The feeling of freshness after use was... (0 = not fresh at all…10 = very fresh)	6.50 ± 2.34	7.21 ± 1.98	4.79 ± 2.99
Number of positive responses Taste of the product			
medical effective cooling soothing astringent others	15 11 11 9 2 5	7 13 14 9 0 9	15 6 6 5 2 7
Taste irritation after usage	Yes: 5 No: 19	Yes: 0 No: 24	Yes: 5 No: 19
Would you use the product regularly?	Yes: 15 No: 9	Yes: 18 No: 6	Yes: 3 No: 21

## Discussion

Since toothbrushing is normally performed twice a day, agents should have – besides their direct anti-plaque effect – good substantivity, defined as the persistence of their activity after application. Agents with high substantivity would preferably remain active and suppress further biofilm growth until the next toothbrushing.^12^

When combining different active substances in toothpastes, complex mutual inactivation of single active substances may occur. Therefore, the entire preparation process should be carefully examined to determine whether single active agents are still effective in the total combination.^3^ Considering all mentioned aspects, the ideal toothpaste should contain active antibacterial agents that would compensate for inadequate mechanical plaque removal by inhibiting remaining dental biofilm and its growth. This study design followed numerous previous studies to test the antibacterial effect and substantivity of agents and formulations in oral hygiene products independent of their mechanical cleaning properties.^3,4,6,8^ The toothpastes were mixed with water, so that simple rinsing reproduced the quantity of active substance present in the oral cavity during normal toothbrushing, without the mechanical cleaning effect. Uncontrolled, methodologically different and often increased toothbrushing by subjects (a typical Hawthorne effect) is avoided. The Hawthorne effect describes a typical phenomenon in toothbrushing studies (but not only), which means that volunteers perform better when they are part of an experiment, or change their behaviour since they receive attention from researchers, rather than due to any manipulation of independent variables. Additionally, the perception of taste can be tested at least similarly (if not better) when toothpastes are already applied in this form.

Since the study was performed analogous to a recent study,^8^ a positive control, i.e. the chlorhexidine solution, could be omitted, which facilitated blinding. In both studies, a toothpaste with sodium bicarbonate/herbal ingredients (SBC) was a comparative product and showed results very similar to those of Arweiler et al,^8^ which confirms the high reproducibility of the test design. Concerning a standard toothpaste with sodium fluoride only as a control (SFL), which was also used in the former study and here revealed clearly lower VF values, it was noticed after unblinding that there were obviously some modifications compared to the previous product. The present study revealed a statistically significant antibacterial and prolonged effect of at least 12 h after a single rinsing with all toothpastes. Both ASC and SFL maintained this statistically significant effect compared to their baseline values, even at the 24-h time point. When comparing groups at all time points, ASC performed better (statistically significantly lower VF values), with the exception of VF_6_, VF_8_ and VF_10_ compared to SFL and VF_10_ compared to SBC. VF_10_ exhibited interesting results, since all three toothpastes revealed very similar VF values. This time point corresponded to a local time between 6 and 8 PM, potentially after dinner, which subjects were allowed to eat. While this was not observed in similar studies,^6,8^ it could be speculated that eating increased the vitality of dental biofilm again in ASC.

Since substantivity studies by other researchers were conducted many years ago and studies with the comparative toothpastes are lacking, it can only be speculated whether the declared active agents themselves or the whole toothpaste formulation accounted for the antibacterial and prolonged effect.

SBC toothpaste based on extracts, such as echinacea, myrrh, rhatany, chamomile, or bicarbonate and sodium bicarbonate statistically significantly reduced VF compared to its baseline, but not to the extent to which ASC did. Values were very similar to that of the control toothpaste (SFL) at all time points. This is in line with previous findings, showing a moderate or low antibacterial effect.^21,22,30^

For the tested control toothpaste with sodium fluoride only (and no specifically declared agents), no substantivity data or recent clinical studies by other researchers could be found. As mentioned above, the lower VF values compared to the earlier study^8^ were remarkable, but it can only be speculated whether the small changes in INCI (International Nomenclature of Cosmetic Ingredients) declaration accounted for it.

ASC is a new toothpaste formulation with a combination of amine fluoride/stannous chloride (1400 ppm fluoride) as well as caprylylglycole and phenylpropanol as relatively new ingredients. Compared to the data of a similar toothpaste – however with amine fluoride/stannous fluoride (MER=ASF) – from an earlier study,^8^ the new formulation showed very similar values that were (also) maintained until VF_24_. Despite the limitations of a direct comparison of the two studies, ASC showed a tendency toward lower VF values at time points 12 and 24 h compared to ASF, while VF_10_ exhibited higher vitality than ASF and, in the present study, the highest vitality after baseline.

So far, this new toothpaste formulation has been tested for its anti-plaque and anti-inflammatory effects in comparison to a monofluorophoshate (MFP) toothpaste in a clinical study where 240 subjects had to brush twice daily at home.^19^ While plaque and gingivitis were statistically significantly reduced by both toothpastes, the new formulation with amine fluoride/stannous chloride led to a clinically relevant and more pronounced plaque reduction compared to the MFP toothpaste.

Besides the assessment of bacterial vitality as the primary outcome and qualitative aspect of biofilms, the plaque area (PA%) was determined as the secondary outcome and quantitative measurement. While the plaque area parameter did not reveal any statistically significant differences between all three toothpastes, it should be kept in mind that all toothpaste slurries were applied on an established biofilm of 48 h and PA was evaluated after a total biofilm growth of 72 h. This parameter was intended to expose a plaque inhibiting effect between the different toothpastes, but failed to do so. A further secondary parameter, patients’ VAS assessment of taste and freshness, pointed out that ASC and SFL were statistically significantly better accepted than SBC. This is in line with the previous study by Arweiler et al,^7^ but again there is no literature to compare these data on acceptance of the three toothpastes. When comparing the previous ASF acceptance data with the those of ASC in this study (as far as possible with completely other subjects), the acceptance of regular use in the future is very similar (ASF 16 yes, 8 no; ASC 15 yes, 9 no), while taste irritation was higher with ASC (5 yes, 19 no) than ASF (1 yes, 23 no).

## Conclusion

All three toothpastes showed a statistically significant antibacterial effect on their baseline values, representing the bacterial vitality of an established 48-h plaque biofilm. This statistically significant effect was continuously present until 12 h after a single rinsing for all toothpastes and after 24 h for ASC and SFL. ASC presented the most pronounced effect, which was superior to SBC at 6 out of 7 time points and to SFL at 3 out of 7 time points.
